# Nectin-2-mediated entry of a syncytial strain of herpes simplex virus via pH-independent fusion with the plasma membrane of Chinese hamster ovary cells

**DOI:** 10.1186/1743-422X-3-105

**Published:** 2006-12-27

**Authors:** Mark G Delboy, Jennifer L Patterson, Aimee M Hollander, Anthony V Nicola

**Affiliations:** 1 Department of Microbiology and Immunology, Medical College of Virginia at Virginia Commonwealth University, Richmond, Virginia, 23298-0678, USA

## Abstract

**Background:**

Herpes simplex virus (HSV) can utilize multiple pathways to enter host cells. The factors that determine which route is taken are not clear. Chinese hamster ovary (CHO) cells that express glycoprotein D (gD)-binding receptors are model cells that support a pH-dependent, endocytic entry pathway for all HSV strains tested to date. Fusion-from-without (FFWO) is the induction of target cell fusion by addition of intact virions to cell monolayers in the absence of viral protein expression. The receptor requirements for HSV-induced FFWO are not known. We used the syncytial HSV-1 strain ANG path as a tool to evaluate the complex interplay between receptor usage, membrane fusion, and selection of entry pathway.

**Results:**

Inhibitors of endocytosis and endosome acidification blocked ANG path entry into CHO cells expressing nectin-1 receptors, but not CHO-nectin-2 cells. Thus, under these conditions, nectin-2 mediates pH-independent entry at the plasma membrane. In addition, CHO-nectin-2 cells supported pH-dependent, endocytic entry of different strains of HSV-1, including rid1 and HFEM. The kinetics of ANG path entry was rapid (t_1/2 _of 5–10 min) regardless of entry route. However, HSV-1 ANG path entry by fusion with the CHO-nectin-2 cell plasma membrane was more efficient and resulted in larger syncytia. ANG path virions added to the surface of CHO-nectin-2 cells, but not receptor-negative CHO cells or CHO-nectin-1 cells, induced rapid FFWO.

**Conclusion:**

HSV-1 ANG path can enter CHO cells by either endocytic or non-endocytic pathways depending on whether nectin-1 or nectin-2 is present. In addition to these cellular receptors, one or more viral determinants is important for the selection of entry pathway. HSV-induced FFWO depends on the presence of an appropriate gD-receptor in the target membrane. Nectin-1 and nectin-2 target ANG path to divergent cellular pathways, and these receptors may have different roles in triggering viral membrane fusion.

## Background

Productive entry of HSV into host cells proceeds following endocytosis [1] or by direct penetration at the cell surface [2]. The viral and cellular factors that determine which pathway is utilized are not clear. The viral envelope glycoproteins gB, gD, and gH-gL are required for entry by both endocytic and non-endocytic routes [3-7]. Expression of a cellular entry receptor is required for both penetration at the plasma membrane and for penetration following endocytosis [1,7-9]. Such receptors function individually and can mediate entry into non-permissive cells, such as Chinese hamster ovary (CHO) cells [10]. The viral ligand for HSV entry receptors is gD [11-17]. In the absence of a gD-receptor, HSV is still endocytosed by CHO cells, but fails to penetrate the endosomal membrane and is degraded [7].

The known gD-receptors include nectins, which belong to a subgroup of the immunoglobulin (Ig) superfamily [17-20]. They are broadly distributed cell-cell adhesion molecules that are components of cadherin-based adherens junctions [21]. Nectin-1 and nectin-2 are ~40% identical, and their N-terminal Ig-like variable (V) domains are critical for gD-binding [11,22-26] and for viral entry [11,23-28]. All HSV strains tested to date [11,17,29] are able to utilize nectin-1 as an entry receptor. Nectin-2 mediates entry of several laboratory strains and clinical isolates of HSV-1 and HSV-2, including HSV-1 isolates from the CNS of patients with herpes simplex encephalitis [19,29]. Amino acid changes in gD at residues 25, 27, or 28 confer the ability to utilize nectin-2 [19,24,30,31]. Additional gD-receptors include HVEM, a member of the TNF-receptor superfamily [10] and heparan sulfate that has been modified by 3-O-sulfotransferase-3 [32]. Nectin-3 [33] and B5 [34] also mediate HSV entry, but their viral ligand(s) is not clear.

Following endocytosis from the cell surface, HSV entry into a subset of cell types also requires intracellular low pH [1,7,9,35,36]. CHO cells expressing gD-receptors are a widely used, well-characterized model system to study pH-dependent, endocytic entry. Inhibitors of endosomal acidification block HSV entry at a step subsequent to endocytic uptake but prior to penetration of the capsid into the cytosol [7]. It has been proposed that HSV utilizes distinct cellular pathways to enter its relevant target cells [35]. Alphaherpesviruses undergo pH-dependent, endocytic entry into certain epithelial cells [1,9,35], including primary human epidermal keratinocytes [35], yet utilize a pH-independent entry pathway into neurons [35,37,38]. Recently, Whitbeck et al. showed that in vitro binding of HSV to liposomes could be triggered by a combination of receptor-binding and low pH [39].

Direct study of the membrane fusion activity of herpesvirions has proven difficult. Fusion-from-without (FFWO) is the induction of target cell fusion by addition of intact virions to the monolayer surface in the absence of viral protein expression. Virus-cell fusion during entry and virion-induced FFWO are analogous inasmuch as both involve similar effector (virion) membranes and target membranes. Several syncytial strains of HSV-1, such as ANG path, are capable of triggering FFWO [40]. HSV-induced FFWO is cell type-dependent [40], but the receptor requirements of FFWO are not known. In the present study, ANG path is used as a tool to investigate the influence of viral and cellular proteins on the route that HSV takes into cells. The ANG path-CHO cell model system allows examination of the inter-relatedness of gD-receptor usage, HSV-induced fusion, and selection of entry pathway.

## Results

### HSV-1 strain ANG path can utilize nectin-1 or nectin-2 for entry into CHO cells

**Figure 1:**
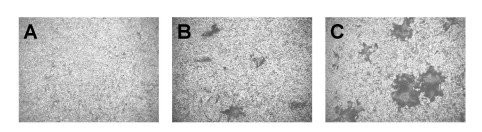
Syncytium formation of HSV-1 strain ANG path on CHO cells expressing nectin-1 or nectin-2. HSV-1 ANG path was added to wild type CHO (A), CHO-nectin-1 (B) or CHO-nectin-2 cells (C) for 24 h. Based on the titration of ANG path on Vero cells, the MOIs were 1000 (A), 100 (B), or 1 (C). Visualization of syncytia was facilitated by immunoperoxidase staining with HR50 antibody to HSV. Magnification, 4×.

First we determined that nectin-1 or nectin-2 can each function to mediate HSV-1 ANG path entry into CHO cells. All strains of HSV-1 and HSV-2 can utilize nectin-1 for entry. The HSV-1 strain ANG path and its parent ANG have alterations in gD at positions 25 and 27 that are predictive of nectin-2 utilization [19,24,41,42]. ANG utilizes both nectin-1 and nectin-2 for entry into CHO cells [17,19]. Monolayers of CHO cells expressing nectin-1 or nectin-2 were infected with serial dilutions of HSV-1 ANG path. As expected, ANG path failed to infect receptor-negative CHO cells (Fig. 1A), but formed syncytia on CHO nectin-1 and CHO-nectin-2 cells (Fig. 1B and 1C). Similar results were obtained using a beta-galactosidase reporter assay for HSV entry (data not shown). The ANG path syncytia that formed on CHO-nectin-2 cells were ~50% larger than those that formed on CHO-nectin-1 cells (Fig. 1B and 1C). The larger plaque size may reflect enhanced entry activity and/or cell-to-cell spread mediated by nectin-2.

### HSV-1 strain ANG path has enhanced plating efficiency on nectin-2 cells relative to nectin-1 cells

Plaque-forming strains of HSV such as KOS and KOS-rid1 do not form substantial plaques on receptor-expressing CHO cells. Hence, to determine the plating efficiency of ANG path we employed the syncytial HSV-1 strain MP [43] for comparison. Unlike many other strains, HSV-1 MP enters receptor-negative CHO cells with low efficiency [10]. The expression of nectin-1, but not nectin-2, enhances MP entry [17,19] in the CHO cell background. MP is not a FFWO strain.

The plating efficiency of ANG path on CHO-nectin-2 cells was approximately two logs greater than on CHO-nectin-1 cells (Table 1). The plating efficiency on CHO-nectin-2 cells was approximately two logs less than that obtained on Vero cells. MP formed syncytia on wild type CHO cells at reduced efficiency (approximately two logs) as compared to Vero cells (Table 1). The presence of nectin-2 did not enhance MP infection above the CHO cell background, but instead reduced the plating efficiency for reasons that are not clear. MP had a 2 log enhanced plating efficiency on CHO-nectin-1 cells relative to CHO-nectin-2 cells (Table 1), which is consistent with previous reports. Importantly, as CHO-nectin-1 cells support MP entry and syncytium formation, the reduced efficiency of ANG path entry is not due to receptor expression levels or some other defect of the CHO-nectin-1 cells. Also in support of this notion, CHO-nectin-1 cells are equivalent to CHO-nectin-2 cells in their ability to support entry of HSV-1 rid1 [29]. Together, the results indicate that ANG path can use either nectin-1 or nectin-2 for entry into the CHO cell lines, but it utilizes nectin-2 more efficiently.

**Table 1:** Plating efficiency of HSV-1 syncytial strains

		**Cell type**		
	Vero	CHO	CHO-nectin-1	CHO-nectin-2

**Virus**		**Titer (PFU/ml)**		

ANG path	8.2 × 10^7^	0	9.8 × 10^3^	1.2 × 10^6^
MP	7.5 × 10^7^	1.9 × 10^4^	7.0 × 10^5^	3.2 × 10^2^

Viruses were titered by limiting dilution. Similar results were obtained in at least three independent experiments.

### HSV-1 ANG path entry mediated by nectin-1 or nectin-2 receptors occurs via distinct cellular pathways

**Figure 2:**
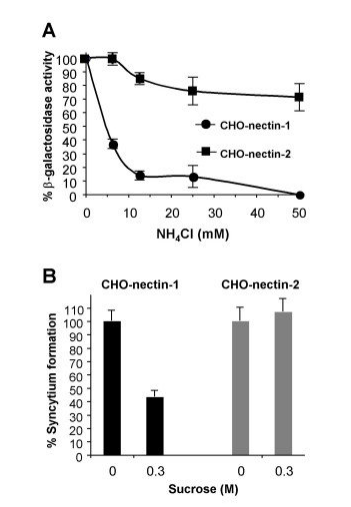
Dependence of ANG path entry on intracellular low pH and endocytosis. (A) Effect of alteration of intracellular pH on ANG path entry. CHO cells expressing either nectin-1 or nectin-2 were treated with the indicated concentrations of ammonium chloride (NH_4_Cl) for 30 min. HSV-1 strain ANG path was added (MOI of 10) for 6 h in the continued presence of NH_4_Cl. Cells contain the *lacZ *gene under the control of an HSV-inducible promoter. Entry was measured as the % of beta-galactosidase activity relative to that obtained in the absence of agent. Data shown are means of quadruplicate determinations with standard deviation. (B) Effect of inhibition of endocytic uptake on ANG path infection. ANG path was added to CHO-nectin-1 or CHO-nectin-2 cells (100 PFU/well) in control medium (A) or hypertonic medium containing 0.3 M sucrose. At 30 min post-infection, medium was removed, extracellular virus was acid-inactivated, and plates were incubated for 24 h. Syncytia were detected by immunoperoxidase staining and quantified. Shown are representative data from at least three independent experiments.

The entry of wild type strains of HSV-1 and HSV-2 into CHO cells expressing gD-receptors is blocked by agents that affect endosome acidification [1], and is consequently considered pH-dependent. Entry of ANG path into CHO-nectin-1 cells was inhibited significantly by the weak base ammonium chloride (Fig. 2A). Surprisingly, entry of ANG path into the nectin-2-expressing cells was refractory to inhibition by the low-pH-altering agents. Similar results were obtained with MOIs ranging from 0.1 to 100 (data not shown). Thus, ANG path stands out as the only HSV strain known to enter a CHO cell line (CHO-nectin-2 cells) by a pH-independent pathway. This suggests that nectin-1 and nectin-2 direct HSV-1 ANG path to distinct entry pathways in the CHO cell.

### ANG path enters CHO-nectin-2 cells by pH-independent fusion with the plasma membrane

The pH-independence of entry does not necessarily indicate entry at the plasma membrane. For example, entry of Epstein Barr virus into B cells is pH-independent, yet it proceeds via an endocytic pathway [44,45]. In addition, Milne et al. demonstrated that HSV enters murine melanoma cells by a pH-independent, endocytic pathway [8]. To assess directly the role of endocytosis, we used cell treatments that selectively block HSV entry by endocytosis. First, we analyzed the effect of high sucrose (hypertonic) medium, which inhibits endocytic uptake of HSV from the plasma membrane, but has no effect on HSV penetration at the plasma membrane [1]. Treatment of CHO-nectin-1 cells with hypertonic medium during virus entry inhibited syncytium formation of HSV-1 ANG path (Fig. 2B). In contrast, hypertonic treatment of CHO-nectin-2 cells had no inhibitory effect (Fig. 2B), suggesting that ANG path penetrates the CHO-nectin-2 plasma membrane in a pH-independent, non-endocytic manner. Thus, deposit of the HSV capsid under the plasma membrane of CHO cells can lead to productive entry.

### CHO-nectin-2 cells can support either endocytic or non-endocytic entry of HSV depending on the virus strain

**Figure 3:**
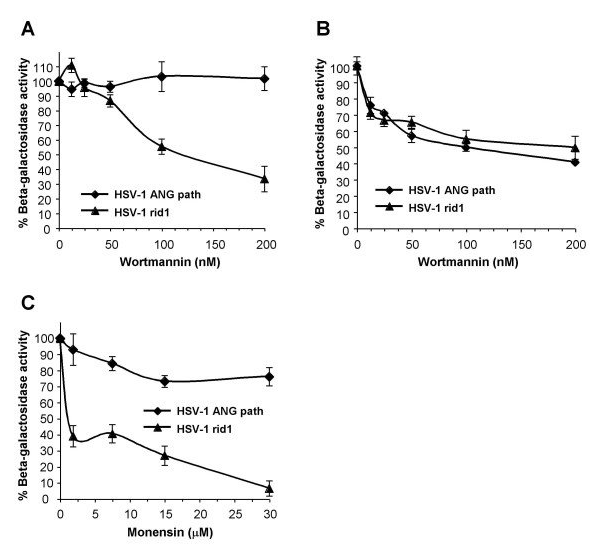
CHO cell entry pathways of HSV-1 rid1 and ANG path mediated by nectin-2. CHO-nectin-2 (A, C) or CHO-nectin-1 cells (B) were treated with the indicated concentrations of wortmannin (A, B) or monensin (C) for 30 min. HSV-1 strains rid1 or ANG path were added (MOI of 1) for 6 h in the continued presence of agent. Entry (beta-galactosidase activity) was measured as in the legend to Figure 2.

The phosphatidyl inositol 3-kinase inhibitor wortmannin selectively inhibits pH-dependent, endocytic entry of HSV [7,9,35,36], possibly at a step involving endosomal trafficking [7]. To study the effect of wortmannin on ANG path entry, we included the HSV-1 strain KOS-rid1 [46] as a control because it also utilizes both nectin-1 and nectin-2 for entry [17,19]. Wortmannin inhibited rid1 entry into CHO-nectin-2 cells, but had little inhibitory effect on ANG path entry into these cells (Fig. 3A). Entry of both ANG path and rid1 viruses into CHO-nectin-1 cells was inhibited by wortmannin in a concentration-dependent manner (Fig. 3B). We also tested treatment of CHO-nectin-2 cells with monensin, a carboxylic ionophore that inhibits endosome acidification. Monensin inhibited rid1 entry into CHO-nectin-2 cells as previously reported [1], but ANGpath entry was refractory to this treatment (Fig. 3C). These results confirm that nectin-1 supports a pH-dependent, endocytic pathway for ANG path, and that nectin-2 supports pH-independent fusion of ANG path with the plasma membrane of CHO cells. As a single cell line, CHO-nectin-2, supports distinct entry pathways for two different HSV-1 strains, this indicates that HSV contains one or more determinants for the selection of entry pathway. Further, receptor-expressing CHO cells can support HSV entry by multiple pathways.

### Rapid entry kinetics of HSV-1 ANG path by either endocytic or non-endocytic pathways

**Figure 4:**
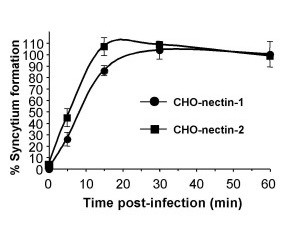
Kinetics of ANG path entry via distinct entry routes. HSV-1 ANG path was bound to CHO-nectin-1 or CHO-nectin-2 cells in 24 well dishes for 1 h at 4°C (100 PFU/well). Cells were washed with PBS and then shifted to 37°C. At the indicated times post-infection, extracellular virus was inactivated by treatment with sodium citrate buffer (pH 3.0). Cells were washed with PBS and incubated for 24 h at 37°C. Cells were fixed in methanol:acetone, and syncytia were quantified by immunoperoxidase staining. Data shown are the mean of quadruplicate samples +/- standard deviation.

The kinetics of entry of a single virus strain by two distinct pathways in the CHO cell background was measured. The entry of ANG path mediated by either nectin-1 or nectin-2 was rapid, with a t_1/2 _of 5–10 min (Fig. 4). By 30 min p.i., greater than 95% of infectious virus had disappeared from the surface of cells regardless of which receptor was present or which pathway was used (Fig. 4).

### ANG path virion-induced fusion of CHO cells is mediated by nectin-2

**Figure 5:**
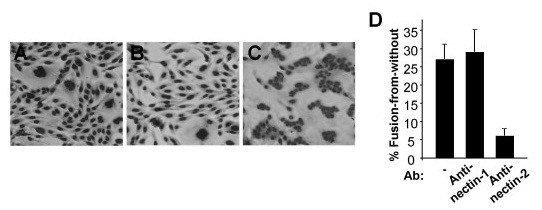
Receptor-dependence of fusion-from-without induced by HSV-1. ANG path virions (MOI of 50) were added to receptor-negative CHO cells (A) or CHO cells expressing either nectin-1 (B) or nectin-2 (C) in the presence of 1 mM cycloheximide. Cells were incubated at 37°C for 3 h, and were then fixed with methanol and stained with Giemsa. Magnification, 4×. Approximately 27% of CHO-nectin-2 cells were fused under these conditions. Results with receptor-negative CHO cells (< 1% fusion) were indistinguishable from CHO-nectin-1 cells. (D) Antibodies to nectin-2 block ANG path-mediated fusion-from-without. Anti-nectin-1 polyclonal antibody R154 or anti-nectin-2 polyclonal antibody R143 was added to CHO-nectin-2 cells in 24 well dishes at 4°C for 30 min. HSV-1 ANG path was added to the monolayers for 37°C for 3 h in the presence of a 1:500 dilution of antibody. Cells were fixed, photographed, and quantified for ANG path-induced FFWO. Experiments were repeated at least three times with similar results.

ANG path is among the subset of syncytial HSV-1 strains that cause fusion-from-without. Addition of ANG path to Vero cells at high multiplicity causes rapid cell fusion (FFWO) in the absence of viral protein synthesis [40,47,48]. Receptor-negative CHO cells are an ideal model system to test the role of gD-receptors. Since ANG path utilizes nectin-2, but not nectin-1, for fusion with plasma membrane during entry, we asked whether nectin-2 would selectively trigger FFWO when ANG path virions were added to the surface of CHO cells. Fusion-from-without was not detected when ANG path virions were added to receptor-negative CHO cells (Fig. 5A). Similarly, FFWO was not detected when ANG path virions were added to CHO-nectin-1 cells (Fig. 5B), even after overnight incubation with an MOI of 1000 (data not shown). However, by 3 h p.i. in the presence of cycloheximide, ANG path virions induced dramatic FFWO of CHO-nectin-2 cells (Fig. 5C). Fusion of cells was evident as early as 30 – 45 min p.i. (data not shown). As there was no viral protein synthesis, it is likely that the viral particles themselves triggered the fusion of cells.

To demonstrate that nectin-2 was specifically responsible for triggering FFWO, CHO-nectin-2 cells were pretreated with antibody to nectin-2 and assessed for fusion. The anti-nectin-2 polyclonal antibody R143 inhibited ANG path virion-induced FFWO of CHO-nectin-2 cells (Fig. 5D). The control anti-nectin-1 antibody R154 had no inhibitory effect on this fusion process (Fig. 5D). Thus, HSV-induced FFWO depends on an appropriate gD-receptor in the target membrane. The results suggest that the ability of nectin-2 to mediate rapid, pH-independent entry at the plasma membrane (Fig. 2 and Fig. 4) correlates with its ability to trigger rapid, pH-independent FFWO (Fig. 5).

### The HSV-1 FFWO strain HFEM does not cause detectable nectin-2 mediated pH-independent fusion

**Figure 6:**
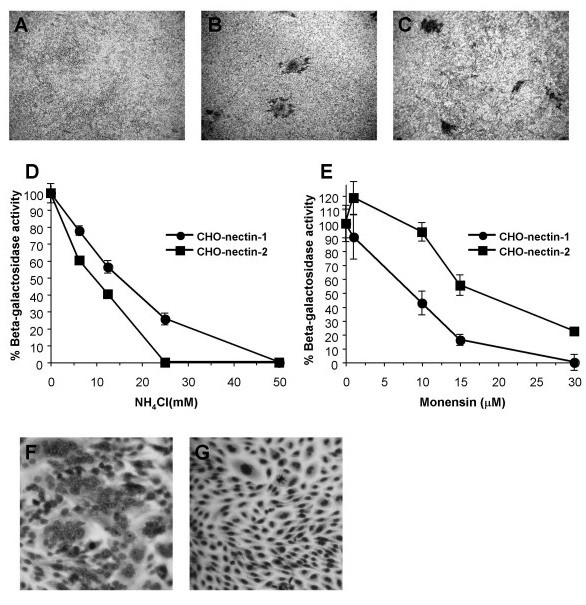
Entry and FFWO activities of HSV-1 strain HFEMsyn. As in the legend to Figure 1, syncytium formation of HFEMsyn was determined on wild type CHO (A), CHO-nectin-1 (B) or CHO-nectin-2 cells (C). Images represent MOIs of 1000 (A, C) or 1 (B). Effect of lysosomotropic agents on HFEMsyn entry. Virus entry into CHO-nectin-1 or CHO-nectin-2 cells in the presence of ammonium chloride (D) or monensin (E) was assayed as in the legend to Figure 2. Cells were infected with HFEMsyn at equivalent multiplicities based on the plating efficiency of HFEMsyn on the respective cell types. Based on Vero cell titer, this corresponds to MOIs of 1 and 450 for CHO nectin-1 and CHO-nectin-2 cells, respectively. Receptor-triggered FFWO of HFEMsyn. As in the legend to Figure 5, HFEMsyn was added to CHO-nectin-1 (F) or CHO-nectin-2 cells (G).

We examined HSV-1 HFEMsyn to determine whether the entry and fusion phenotypes of ANG path were shared by another strain. Like ANG path, HFEMsyn has a syncytial phenotype and causes FFWO [49]. Receptor-negative CHO cells were refractory to infection by HSV-1 HFEMsyn (Fig. 6A). Both CHO-nectin-1 cells and CHO-nectin-2 cells supported syncytium formation by HFEMsyn (Fig. 6B and 6C). HFEMsyn utilized nectin-2 three logs less efficiently than nectin-1. HFEM entry into either CHO-nectin-1 or CHO-nectin-2 cells was inhibited by both ammonium chloride and monensin (Fig. 6D and 6E), indicating pH-dependent entry in both cell types. ANG path may have a unique determinant that enables entry by fusion with the plasma membrane of CHO-nectin-2 cells.

Unlike ANG path, HFEMsyn triggered detectable FFWO on the CHO-nectin-1 cells, but not the CHO-nectin-2 cells (Fig. 6F and 6G). Nectin-1 can thus trigger HSV-induced FFWO. The results suggest that FFWO does not correlate with plasma membrane fusion during entry. Instead, the ability of a FFWO strain to efficiently utilize a given receptor may correlate with its ability to cause FFWO triggered by that receptor.

## Discussion

A given animal virus can enter cells by multiple pathways [50]. HSV can enter its host cells by endocytosis or by direct penetration at the plasma membrane. How a particular pathway is selected is of fundamental importance. CHO cells that express gD-receptors support pH-dependent, endocytic entry of HSV. We identified a laboratory strain of HSV-1, ANG path, that can enter CHO cells by pH-independent fusion with the plasma membrane in a receptor-specific manner. Our results indicate that gD-receptors are required for FFWO. Viral determinants, cellular gD-receptors, and the background of the target cell all contribute to the entry route taken by HSV.

### Host cell determinants of HSV entry pathway

Previous studies have indicated a role for the target cell in determination of HSV entry pathway [1,8,9,35]. Murine melanoma cells are non-permissive for HSV entry. Expression of a gD-receptor results in endocytic uptake of HSV from the cell surface and subsequent pH-independent penetration from an endosome [8]. In contrast, initial endocytic uptake from the surface of CHO cells occurs independently of the known gD-receptors [7]. CHO cells may contain unidentified cellular receptors needed for internalization of HSV from the surface. BHK-derived, J cells that express nectin-1 support pH-independent entry of HSV [9]. Fusion of nectin-1 with either carboxy-terminal sequences of epidermal growth factor or with a glycosylphoshatidylinositiol anchor resulted in chimeric receptors that support pH-dependent entry into J cells [9]. Thus, alternate forms of nectin-1 can mediate different entry routes.

The current study indicates that nectin-1 and nectin-2 differ functionally in their ability to target incoming ANG path virions in CHO cells. These receptors interact with distinct yet overlapping regions of gD [19,24,30,31,51]. In our experimental system, nectin-1 and nectin-2 may mediate pH-dependent and pH-independent membrane fusion, respectively. We are currently investigating the receptors and entry pathways that ANG path utilizes in other target cells.

### Viral determinants of HSV entry pathway

HSV contains one or more determinants for the selection of entry pathway (Fig. 3). Candidate determinants include gB, gD, and gH-gL which are essential for entry [3-6]. Compared to the wild type HSV-1 KOS strain, the gB [47] and gD [42] of ANG path have 10 and 7 amino acid differences, respectively. Alterations in gD at positions 25 and 27 [52] as well as ectodomain and cytoplasmic tail mutations in gB [47,48] have been proposed to be important for FFWO activity. The role of these residues in the selection of entry route is currently being evaluated.

The composition of the ANG path virion allows direct triggering of fusion by nectin-2, at least in the context of the CHO cells tested. One possibility is that ANGpath interaction with nectin-2 is sufficient to functionally substitute for the combination of nectin-1 interaction and exposure to intracellular low pH. Analysis of the difference between these receptor interactions may lead to a better understanding of how membrane fusion is triggered during HSV entry. Interestingly, ANG path entry into Vero cells is also unique in that it is highly resistant to inhibition by soluble, ectodomain forms of gD [53].

### Fusion-from-without as a model for membrane fusion during HSV entry

A current model of HSV entry posits that gD binding to receptor triggers a cascade of events culminating in fusion [54-57]. The viral and cellular requirements for HSV entry have been largely recapitulated in a cell-to-cell fusion assay [31,58-66]. In this surrogate assay, transfected cells that express gB, gD, gH and gL on the cell surface are mixed with untransfected target cells. Comparisons of cell-cell fusion with virus-cell fusion must be drawn cautiously. Herpesviral envelopes are derived from internal cellular membranes, not the plasma membrane. It is possible that glycoproteins displayed on the plasma membrane of transfected cells have distinct roles in fusion (i.e., are activated differently) than glycoproteins that are actually incorporated into virions.

FFWO is an underutilized model to analyze the membrane fusion activity of HSV particles. Although a high MOI is required to detect FFWO, virus-cell fusion during entry and FFWO have significant similarities. The effector membrane and target cell membrane are analogous for both fusion processes. Furthermore, FFWO, like HSV entry, is gD-receptor dependent.

## Conclusion

Two members of the nectin family of HSV receptors, nectin-1 and nectin-2 can target the same laboratory strain of HSV to endocytic and non-endocytic pathways, respectively. The combination of ANGpath and nectin-2 at the surface of a CHO cell line triggers rapid, pH-independent membrane fusion that can lead to viral entry or FFWO. An appropriate gD-receptor is required for HSV-induced FFWO. This is similar to the receptor requirement for the membrane fusion processes that accompany viral entry or cell-to-cell fusion. Together, the results indicate that viral factors, in addition to cellular factors such as nectins, contribute to the selection of HSV entry route. This report demonstrates that the ANG path-CHO cell system can serve as a model to study the molecular connections between receptor usage, membrane fusion, and choice of entry pathway.

## Methods

### Cells and viruses

Vero cells (American Type Culture Collection; ATCC; Rockville, Md.) were propagated in Dulbecco's Modified Eagle's Medium (Invitrogen, Grand Island, NY) supplemented with 8% fetal bovine serum (FBS; Gemini Bio-Products, West Sacramento, Calif.). CHO-K1 cells stably transformed with the *Escherichia coli *lacZ gene under the control of the HSV ICP4 promoter are designated CHO IEβ8 [10]. CHO IEβ8 cells stably transformed to express nectin-1 (M3A cells) or nectin-2 (M2A cells) [13,17,19] (provided by G. Cohen and R. Eisenberg, University of Pennsylvania) were propagated in complete medium, Ham's F12 nutrient mixture (Invitrogen) supplemented with 10% FBS, 150 μg/ml puromycin (Sigma, St. Louis, Mo.), and 250 μg/ml G418 sulfate (Fisher Scientific, Fair Lawn, NJ). 100% of cells expressed nectin-1 or nectin-2 on the cell surface as determined by immunofluorescence. Cells were subcultured in non-selective medium prior to use in all experiments.

HSV-1 strains ANG path [67] and KOS were obtained from T. Holland, Wayne State University. HSV-1 MP [43] was obtained from ATCC. HSV-1 HFEM [68] and KOS-rid1 were obtained from P. Spear, Northwestern University. Rid1 is a KOS derivative with a Q27P mutation in gD [46]. Virus stocks were grown and titered on Vero cells.

### Plaque assay

At 18 – 24 h p.i. culture medium was removed, and cells were fixed with ice-cold methanol-acetone solution (2:1 ratio) for 20 min at -20°C and air-dried. Virus titers or syncytium formation were determined by immunoperoxidase staining with anti-HSV polyclonal antibody HR50 (Fitzgerald Industries, Concord, Mass.).

### Beta-galactosidase reporter assay for HSV entry

Confluent cell monolayers grown in 96 well dishes were infected with HSV-1 and incubated at 37°C for 6 h. 0.5% Nonidet P-40 (Sigma) cell lysates were prepared, chlorophenol red-b-D-galactopyranoside (Roche Diagnostic, Indianapolis, In.) was added, and beta-galactosidase activity was read at 595 nm with a microtiter plate reader (BioTek Instruments, Winooski, Vt.). Mean results and standard deviations were calculated for four replicate samples.

### Inhibition of uptake from cell surface

HSV was prebound to cells in 24 well dishes (100 PFU/well) in culture medium containing 20 mM HEPES and 0.2% BSA at 4°C for 2 h. Cells were treated with medium containing 0.3 M sucrose (hypertonic), or control complete medium for 30 min at 37°C. Cells were washed with phosphate buffered saline **(**PBS), and the remaining surface-bound virions were inactivated by sodium citrate buffer (pH 3.0) for 2 min at 37°C. Cells were incubated in normal medium for 24 h, and then syncytia were quantified.

### Treatments with lysosomotropic agents

Performed as reported previously [1]. Briefly, cells were treated with medium containing ammonium chloride or monensin for 30 min at 37°C. Virus was added, and cells were incubated in the constant presence of agent for 6 h. Beta-galactosidase activity indicated successful entry.

### Virion-induced fusion-from-without assay

Confluent cell monolayers grown in 24 or 96 well dishes were pretreated with growth medium containing 0.5 mM cycloheximide (Sigma) for 15 min. Cell-free supernatant preparations of HSV-1 ANG path were added to cells at multiplicities from 1 to 500 for up to 3 h at 37°C in the constant presence of cycloheximide. Cells were rinsed with PBS, and then fixed in 100% methanol. Monolayers were air dried, and then nuclei were stained with Giemsa.

To measure inhibition of fusion by anti-receptor antibodies, cells were chilled to 4°C for 10 min. Rabbit polyclonal serum against nectin-1 (R154) or nectin-2 (R143) (obtained from R. Eisenberg and G. Cohen, University of Pennsylvania) were added for 30 min at 4°C at a 1:500 dilution in culture medium adjusted to 20 mM HEPES. HSV-1 ANG path was added, and plates were incubated for 3 h at 37°C in the presence of antibody.

Micrographs were taken with a Zeiss Axiovert 40C microscope equipped with a Canon PowerShot G6 digital camera. Digital images were processed with Adobe Photoshop CS2 version 9.0. To quantitate fusion, photomicrographs of random fields from triplicate wells (> 500 cells/well) were scored. The number of nuclei present in clusters of 5 or more divided by the total number of nuclei yielded the % fusion.

## Competing interests

The author(s) declare that they have no competing interests.

## Authors' contributions

MD carried out HSV-1 rid1, HFEM, and wortmannin analyses. JP carried out fusion-from-without studies. AH performed energy depletion experiments. AN conceived of the study, carried out plating efficiency, kinetics, and lysosomotropic agent experiments, and supervised the work.
